# Response to the publication by Landrigan PJ, Straif K. Aspartame and cancer – new evidence causation

**DOI:** 10.1186/s12940-021-00788-x

**Published:** 2021-09-21

**Authors:** Ashley Roberts

**Affiliations:** AR Toxicology Inc., Oakville, Canada

A recent re-evaluation of the information regarding aspartame and cancer from studies conducted at the Ramazzini institute (RI) was published within Environmental Health [1]. This publication referenced additional analysis conducted by RI regarding aspartame induced haematopoietic and lymphoid tumours (HLT’s) in rats after life-time treatment [2]. The lesions originally diagnosed as lymphoma or leukemia were re-examined and reclassified according to the INHAND Criteria [3].

In response, a thorough review was conducted of the 2 latest RI articles combined with previous regulatory opinions, evidence from the large aspartame safety data base and dietary intake analysis. Following evaluation of the histological slides presented within the Tibaldi *et al* 2020 publication, the immunoblastic lymphoma and lymphoblastic lymphoma lesions were determined to be composed of small separate (or multifocal) aggregations, which were accompanied by infiltration of macrophages and neutrophils. Using INHAND nomenclature and diagnostic criteria for chronic lung inflammation (see below), these lesions are therefore considered to result from chronic inflammation [3]. INHAND’s diagnostic features of chronic inflammation are “perivascular and peribronchiolar mononuclear inflammatory cells”, whereas the diagnostic feature of neoplasia is described as “uniform cell population infiltrating entire section”. This viewpoint is corroborated through the identification of interstitial fibrosis within the histological images, which is described by INHAND as a diagnostic feature of chronic lung inflammation [3]. Furthermore, INHAND’s description of lymphoma within the haematopoietic system indicates that “the diagnosis of a single site tumour can be difficult and is based on abnormal cytological features, disturbance of tissue architecture and absence of inflammation and infection”. The slide images show no clear evidence of any disturbance of tissue architecture while clear evidence of inflammation is observed. Furthermore, while the authors claim that tumour cells were found in multiple organs, evidence of this was not reported in the manuscript. In addition, while Landrigan and Straif report that no mycoplasma infection was detected in the rat study, no evidence to support this or any other microbial infection was presented [2]. In addition, lymphocytic lymphoma does not have a high degree of cell atypia and is largely indistinguishable from normal lymphocytes [4]. Confirmation of whether the lesion is a tumour is therefore only possible through comparison with surrounding tissues and the extent of the lesion. Such a comparison with surrounding tissues was missing from the evaluation. Furthermore, INHAND’s description of lymphoid hyperplasia, indicates “increased lymphocyte cellularity (lymphoid hyperplasia) can occur under a variety of circumstances which can either be specific, that is antigen driven by pollutants, particulates, tissue damage, or drainage of inflammation from a distant site”, therefore since severe inflammation lesions were reported in animals within this study, it is therefore considered probable that the lesions arose through chronic inflammation in other organs as a consequence of lymph node hyperplasia. It was also informative, that the authors indicated that some lesions initially diagnosed as lymphoma or leukemia were in fact chronic inflammatory lesions accompanied by proliferation of fibroblasts [2]. Moreover, EFSA [5, 6] had noted that the treatment effects were not significantly different from historical controls in their original studies. Tibaldi *et al* 2020 does not address on how the treatment effect on HLT compare to historical controls.

The re-analysis of those tumours originally identified within the RI, therefore failed to adequately address the concerns raised by International Regulatory Authorities, including EFSA [5, 6] and the U.S.FDA [7] who concluded that the “increased incidence of lymphomas/leukemias reported in treated rats was unrelated to aspartame, given the high background incidence of chronic inflammatory changes in the lungs and the lack of a positive dose-response relationship”. Subsequently the EFSA undertook a re-evaluation of aspartame and concluded “that there were no safety concerns at the current ADI of 40mg/kg bw/day and that using “conservative” estimates of exposure of the general population (95^th^ percentile) the intake was below the ADI.

In conclusion, a close examination of the articles published by RI [1, 2] who re-evaluated the HLT’s from the original life-time aspartame rat studies [8–10], and using INHAND criteria, provided insufficient evidence to challenge and contradict the opinions of both EFSA and the FDA regarding the “shortcomings and uncontrolled variables”, within the study “such as the presence of infection in the test animal”. For lymphocytic lymphoma, the histological images proved to be inconclusive since the relationship with surrounding tissues was not provided and the absence of inflammation in other organs was not reported. Therefore, the lesion is likely a consequence of lymph node hyperplasia following inflammation in the lungs. Finally, no conclusive evidence was provided to substantiate that there was no mycoplasma or other microbial infection within these animals. Overall, the re-evaluation of the HLT’s fails to address the significant “shortcomings” in the RI study and the impact of infection within these animals, and the new data only reinforces the original role of infections and inflammation in the effects reported.

## Data Availability

Not applicable.
